# Diverse Sphingolipid Profiles in Rectal and Colon Cancer

**DOI:** 10.3390/ijms241310867

**Published:** 2023-06-29

**Authors:** Adam R. Markowski, Agnieszka U. Błachnio-Zabielska, Karolina Pogodzińska, Anna J. Markowska, Piotr Zabielski

**Affiliations:** 1 Department of Internal Medicine and Gastroenterology, Polish Red Cross Memorial Municipal Hospital, 79 Henryk Sienkiewicz Street, 15-003 Bialystok, Poland; 2 Department of Hygiene, Epidemiology and Metabolic Disorders, Medical University of Bialystok, 2C Adam Mickiewicz Street, 15-222 Bialystok, Poland; 3 Department of Medical Biology, Medical University of Bialystok, 2C Adam Mickiewicz Street, 15-222 Bialystok, Poland

**Keywords:** colorectal cancer, colon cancer, rectal cancer, ceramide, sphinganine, sphingosine-1-phosphate, sphingolipids, individualized cancer therapy

## Abstract

Colorectal cancer is a heterogenous group of neoplasms showing a variety of clinical and pathological features depending on their anatomical location. Sphingolipids are involved in the formation and progression of cancers, and their changes are an important part of the abnormalities observed during carcinogenesis. Because the course of rectal and colonic cancer differs, the aim of the study was to assess whether the sphingolipid profile is also different in tumors of these two regions. Using a combination of ultra-high-performance liquid chromatography combined with triple quadrupole mass spectrometry, differences in the amounts of cellular sphingolipids were found in colorectal cancer. Sphingosine content was higher in rectal cancer than in adjacent healthy tissue, while the content of two ceramides (C18:0-Cer and C20:0-Cer) was lower. In colon cancer, a higher content of sphingosine, sphinganine, sphingosine-1-phosphate, and two ceramides (C14:0-Cer and C24:0-Cer) was found compared to healthy tissue, but there was no decrease in the amount of any of the assessed sphingolipids. In rectal cancer, the content of sphinganine and three ceramides (C16:0-Cer, C22:0-Cer, C24:0-Cer), as well as the entire pool of ceramides, was significantly lower compared to colon cancer. The S1P/Cer ratio in rectal cancer (S1P/C18:1-Cer, S1P/C20:0-Cer, S1P/C22:0-Cer, S1P/C24:1-Cer) and in colon cancer (S1P/C18:0-Cer, S1P/C18:1-Cer, S1P/C20:0-Cer) was higher than in adjacent healthy tissue and did not differ between the two sites (rectal cancer vs. colonic cancer). It seems that the development of colorectal cancer is accompanied by complex changes in the metabolism of sphingolipids, causing not only qualitative shifts in the ceramide pool of cancer tissue but also quantitative disturbances, depending on the location of the primary tumor.

## 1. Introduction

Colorectal cancer (CRC) is a diverse group of tumors, depending on anatomical location, that display miscellaneous clinical and pathological features. Rectal cancer occurs in 33.03% of men and 25.87% of women [1]. Probably because of the prior appearance of symptoms, depending on, among others, its proximity to the anus, rectal cancer (RC) is detected at an early stage more frequently than colon cancer (CC) [1].

In addition to tumor grading and staging, some genetic, biochemical, and histological markers have been used for the prognostication and optimization of adjuvant treatment [2,3], but there is no consensus on their exact role in RC patients. The accumulation of sphingolipids (SL) accounts for almost 90% of the changes in the colon cancer cell line [4]. The central active molecules in SL metabolism are ceramides (Cer) and their content in the cell depends on the balance between the rate of their production and degradation [5]. Cer are bioactive lipids that consist of a sphingoid base (one of several) and a fatty acyl chain of various lengths and a varied degree of saturation. To date, more than 200 structurally distinct molecular Cer species have been described in mammalian cells, with different biological properties depending on the chemical structure [2]. Cer are relevant components of cell membranes, and in response to stress stimuli, they undergo rapid turnover, which firmly changes their biophysical properties. Furthermore, Cer participate in cell migration and adhesion, proliferation, differentiation, growth inhibition, and apoptosis as major mediators. Growing evidence suggests that Cer are also involved in cancer’s rise and progression. Defects of Cer turnover in cancer cells can favor their continued augmented proliferation and propensity to metastasize. The attainable data indicate that Cer metabolism is modified in numerous cancers [6] and it is suggested that Cer may thus inhibit cancer progression [7]. However, the exact mechanism of such activity is still undetermined. Measurements of Cer content in CRC are inconsistent; studies have shown both lower [4] and higher [3] SL amount in tumor compared to normal mucosa. Cer accumulation is observed after radiation treatment or chemotherapy, and lymph node invasion positively correlated with C24:0-Cer levels in CRC [8].

It is well known that a raised sphingosine-1-phosphate/ceramide ratio (S1P/Cer) is connected with increased colon cancer cell survival, excessive proliferation, and tumor progression [9]. Sphingosine-1-phosphate (S1P) is pro-proliferative agent, while Cer and sphingosine (Sph) are generally pro-apoptotic compounds [3]. Recent findings suggest that modulation of the S1P/Cer ratio regulates both cellular apoptosis and CRC metastasis through Cathepsin-D modulation [10]. On the other hand, long-chain and very-long-chain Cer may have the opposite effect on CRC and a disequilibrium between Cer with various fatty acyl chain lengths could be crucial for cancer progression [11]. Moreover, it seems that a deficiency of some Cer can be compensated for by the increased synthesis of other Cer under certain conditions [3].

Since the course of cancer varies depending on the anatomical site (rectum vs. colon), the aim of the study was to assess whether the location of the tumor affects the SL content and composition in CRC.

## 2. Results

### 2.1. Patients

Of the 30 patients, 24 (80%) were male. The mean age of the patients at diagnosis was 67 years (range 35–90). A total of 53.33% of CRC (n = 16) were located in the rectum, and 46.67% (n = 14) in the colon. The average age of the patients with RC and CC was 68.38 ± 3.55 years (range 35–90) and 65.57 ± 2.79 years (range 48–79), respectively, and did not differ statistically between the groups (*p* = 0.54). The mean tumor size at these two locations was 50.44 ± 6.18 mm and 42.36 ± 5.11 mm, respectively, and was not statistically different between groups (*p* = 0.33). Distant metastases were found in three patients and most patients (n = 19) had no evidence of lymph node metastases.

### 2.2. Content of Sphingolipids in Colorectal Tissue

SL content was expressed as pmol per 1 mg of tissue. Among the evaluated SL, the highest content in CRC was found for long-chain Cer with C16:0 N-acyl chain, i.e., C16:0-Cer (126.75 ± 10.19 pmol/mg), which accounted for 81.05% of the total ceramide (TCer). The second highest SL content in the tumor, although much lower than the leading C16:0-Cer, was very-long-chain ceramide, C24:1-Cer (14.00 ± 0.96 pmol/mg, 8.95% of the total ceramide).

The amount of C16:0-Cer and C24:1-Cer in CRC and in healthy tissue surrounding the tumor was not statistically different (*p* = 0.12 and *p* = 0.71, respectively) (Table 1A). Simultaneously, of the remaining SL, a higher content in CRC than in normal tissue was demonstrated for C14:0-Cer (*p* = 0.003), C24:0-Cer (*p* = 0.0027), Sph (*p* = 0.0001), S1P (*p* = 0.0006), and sphinganine (SPA) (*p* = 0.0003). A lower content in the tumor than in the healthy tissue was found for C18:0-Cer (*p* = 0.036) and C20:0-Cer (*p* = 0.002). In addition, TCer content was statistically the same in the tumor and in the adjacent tissue (*p* = 0.13) (Table 1A).

The number of patients in each group according to the TNM classification was as follows: n = 8 (TNM I), n = 9 (TNM II), n = 10 (TNM III), and n = 3 (TNM IV). In such groups, no statistical differences were found between the analyzed parameters. When patients were divided into two groups (TNM-I+II vs. TNM-III+IV) with a larger number (n = 17 vs. n = 13) of subjects, a significant statistical difference was found in the content of C18:0-Cer in colorectal cancer, namely the level of C18:0-Cer in the TNM-I+II group was lower than in the more advanced TNM-III+IV group (2.807 ± 0.26 vs. 3.727 ± 0.28; *p* = 0.026). A similar trend, which however did not reach statistical significance, was shown for C20:0-Cer. The C20:0-Cer content in the TNM-I+II group was non-significantly lower than in the TNM-III+IV group (0.801 ± 0.1 vs. 1.069 ± 0.10; *p* = 0.076). None of the assessed parameters was gender-dependent.

### 2.3. Content of Sphingolipids in the Tumor Tissue Depending on the Location: Rectum vs. Colon

Taking into account the location of the tumor, other interesting relationships were observed. The content of C18:0-Cer (*p* = 0.027) and C20:0-Cer (*p* = 0.009) in RC was lower than in the surrounding tissue, and the amount of Sph (*p* = 0.005) was higher (Table 1B). The content of other SL in the tumor was not statistically different from the amount in the healthy tissue, although a downward trend was also observed for C22:0-Cer (*p* = 0.071). In CC, the content of several SL was higher than in the adjacent healthy tissue. Such relationships were found for C14:0-Cer (*p* = 0.003), C24:0-Cer (*p* = 0.0001), Sph (*p* = 0.0006), S1P (*p* = 0.001), and SPA (*p* = 0.0001) (Table 1C). The amount of remaining SL was the same in the tumor and surrounding tissue, although an upward trend was seen for C16:0-Cer (*p* = 0.006), C20:0-Cer (*p* = 0.007), and TCer (*p* = 0.057).

**Figure 1:**
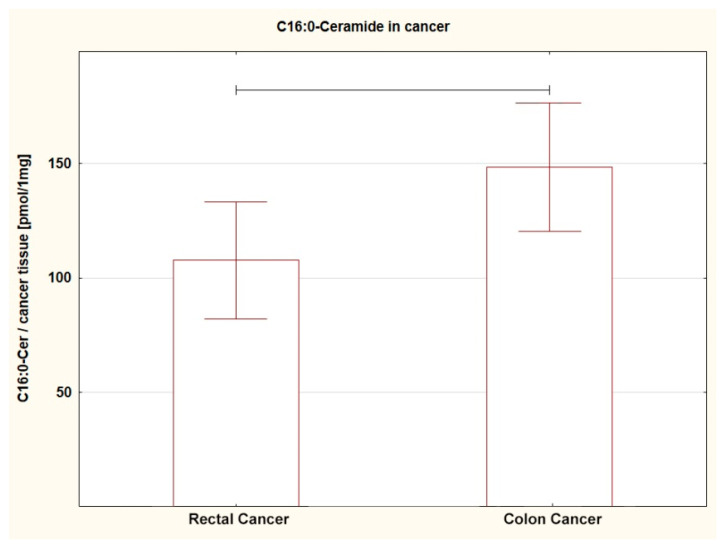
Box plots representing content of C16:0-Ceramide in rectal and colon tumors. The large rectangles demonstrate the mean and the whiskers represent the standard error of mean. *p* = 0.044.

**Figure 2:**
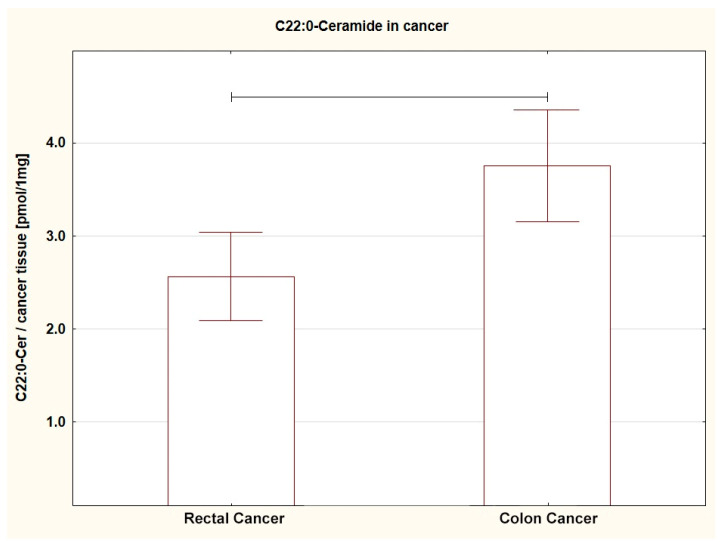
Box plots representing content of C22:0-Ceramide in rectal and colon tumors. The large rectangles demonstrate the mean and the whiskers represent the standard error of mean. *p* = 0.005.

**Figure 3:**
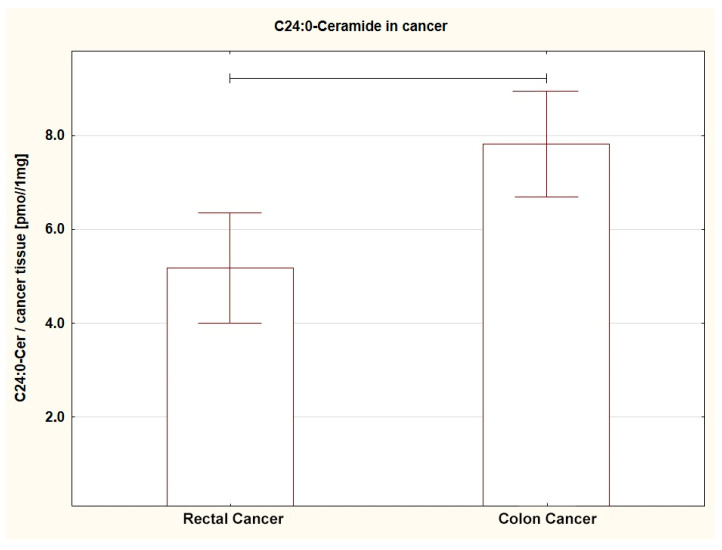
Box plots representing content of C24:0-Ceramide in rectal and colon tumors. The large rectangles demonstrate the mean and the whiskers represent the standard error of mean. *p* = 0.004.

**Figure 4:**
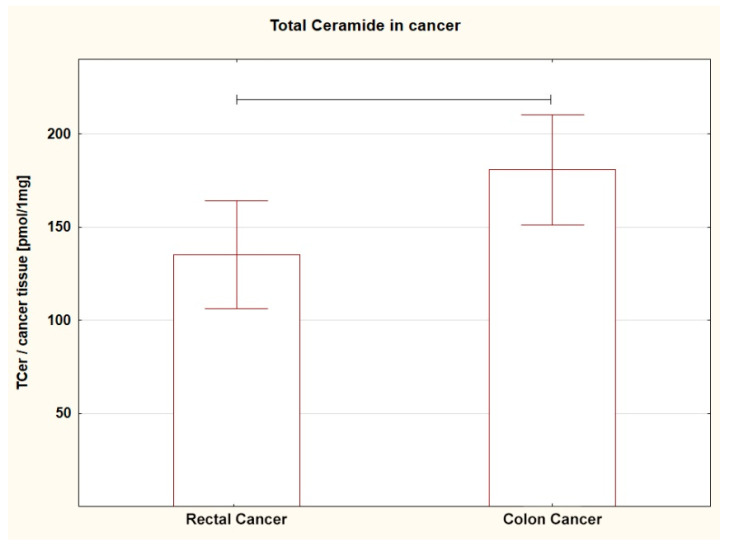
Box plots representing content of total ceramide in rectal and colon tumors. The large rectangles demonstrate the mean and the whiskers represent the standard error of mean. *p* = 0.041.

Comparing the SL content in both tumors (RC vs. CC), a different amount of several SL was found. The level of SPA (*p* = 0.013), C16:0-Cer (*p* = 0.044), C22:0-Cer (*p* = 0.005), and C24:0-Cer (*p* = 0.004) in RC was lower; a similar trend was shown for C14:0-Cer (*p* = 0.053) (Table 2A, Figure 1, Figure 2, Figure 3 and Figure 4). For the remaining SL, the amount was unchanged, but the TCer pool was smaller in RC (*p* = 0.041). Interestingly and importantly, the analysis of the SL content in the tissue adjacent to the cancer did not show any changes in any location, i.e., it was the same in the rectum and colon (Table 2B).

### 2.4. Sphingosine-1-Phosphate to Ceramide Ratio in Colorectal Tissue

We calculated the S1P/Cer ratio for each Cer and compared its value (CRC vs. healthy colorectal tissue). We found statistically significant differences for six ceramide species, and in each case, the S1P/Cer ratio was higher in CRC than in normal tissue: S1P/C18:1-Cer, S1P/C18:0-Cer, S1P/C20:0-Cer, S1P/C22:0-Cer, S1P/C24:1-Cer, and S1P/C24:0-Cer (Table 3A). In addition, we checked the same relationships separately in two locations and found that they were mostly similar. For rectal cancer, the differences (RC vs. healthy rectal tissue) concerned four ceramides: S1P/C18:1-Cer, S1P/C20:0-Cer, S1P/C22:0-Cer, and S1P/C24:1-Cer, although an upward trend was demonstrated for S1P/C24:0-Cer (*p* = 0.097), (Table 3B). As with colon cancer, the differences (CC vs. healthy colon tissue) concerned three ceramides: S1P/C18:0-Cer, S1P/C18:1-Cer, and S1P/C20:0-Cer, although an upward trend was also shown for S1P/C24:1-Cer (*p* = 0.098) (Table 3C). In both tumor locations, the strongest difference between cancer and healthy tissue was noted for S1P/18:1-Cer. There were no differences for S1P/Cer ratios separately between tumors (RC vs. CC) (Table 3D) and between normal tissues from both sites (healthy rectal tissue vs. healthy colon tissue) (Table 3E).

## 3. Discussion

Sphingolipid metabolic pathways in cancer cells are complex and not yet fully understood. In the present study, we evaluated the profile of long-chain and very-long-chain ceramides in colorectal cancer and healthy tissue surrounding a tumor.

Rectal and colon cancer are diverse clinical entities. Cancer risk factors are different in these two locations; family history is more strongly associated with the risk of CC than RC [12] and alcohol consumption is more strongly associated with the risk of RC than CC [13]. In addition, the course of colorectal cancer varies depending on the anatomic location. T1-stage RC patients have a higher rate of lymph node metastases (compared with CC patients) [14], less favorable recurrence outcomes [15], and a higher recurrence rate (regardless of the treatment strategy) [16]. Primary CRC location was not associated with the incidence of brain metastases, but RC patients have a higher incidence of bone [17] and lung metastases [18], and worse relapse-free survival [19]. The development time for RC may take 10 years, but the time between diagnosis and treatment, although relatively short, seems particularly important as the risk of metastasis may be the highest in the last few weeks before surgery [20]. The optimal therapy for patients with rectal cancer is highly individualized, and in advanced stages, may require radiotherapy, chemotherapy, and surgery.

Using a combination of ultra-high-performance liquid chromatography coupled with triple quadrupole mass spectrometry, differences in the amounts of cellular SL were found in CC and RC, suggesting a heterogeneity of SL metabolism in both of these locations. Among the examined SL in CRC (CC and RC analyzed together), the highest content was found for two Cer species with different acyl chain lengths (C16:0-Cer and C24:1-Cer), and it was similar to the amount in healthy bowel tissue. In addition, the TCer content was statistically the same in the tumor and in healthy adjacent tissue. However, the content of some sphingolipids (Sph, SPA, S1P, C14:0-Cer, C24:0-Cer) was higher in CRC. On the other hand, some ceramides (C18:0-Cer and C20:0-Cer) showed a lower content in the tumor than in healthy adjacent tissue. The results of another study conducted at a different center confirm our observations because they revealed that the level of some ceramide species (C16:0-Cer, C24:0-Cer, and C24:1) is increased, while the level of other ceramides (C18:0-Cer and C20:0-Cer) is reduced in CRC [8].

The comparison of SL content in tumors detected in two different locations separately (RC vs. CC) resulted in other interesting findings. The content of C18:0-Cer and C20:0-Cer in RC was lower, and the Sph content was higher than in the healthy tissue. In CC, in addition to an increase in Sph content, a higher amount of other sphingolipids (S1P, SPA, C14:0-Cer, and C24:0-Cer) was additionally found compared to healthy tissue, but no simultaneous decrease in the content of any of SL was observed.

Our observations may help explain the worse course of RC than CC. Thus, for the first time, we showed various distribution trends and interesting relationships regarding the content of SL in CRC compared to adjacent tissue, depending on the anatomical location of the tumor. This was not a random and insignificant find, because such relationships for these two different anatomical locations (rectum vs. colon) were not demonstrated in the surrounding healthy tissue; in normal tissue, there was no difference in the content of any of the sphingolipids, neither in the rectum nor in the colon. Whereas CC showed no decrease in the content of any SL (compared to healthy tissue), RC showed a lower content of two ceramide species: C18:0-Cer and C20:0-Cer. CC, on the other hand, showed an increase in several types of sphingolipids compared to healthy tissue surrounding the tumor (Sph, S1P, SPA, C14:0-CerC24:0-Cer), while rectal cancer showed an increased amount of only one sphingolipid (Sph), and it was one of those that was also higher in colon cancer. In addition, the different distribution of individual Cer species was not accompanied by changes in the content of the TCer pool, which underlines the importance of a qualitative shift rather than quantitative modifications in cancerogenesis.

However, we saw the most interesting changes after comparing the content of sphingolipids directly in RC and CC. We found that in RC, there is a significantly lower content of SPA and three Cer (C16:0-Cer, C22:0-Cer, C24:0-Cer), and the degree of this change is so profound that it also caused a significant decrease in the amount of the entire ceramide pool compared to CC. Based on these results, it can be concluded that the development of cancers in some anatomical locations (e.g., in the rectum) undergoes more complex changes in the ceramide profile, causing not only qualitative shifts but also quantitative changes. The observed changes in the SL profile may be the reason for the worse course of RC. This suggestion seems to be supported by another study that showed that SPA (lower in RC) stopped the cell cycle, inhibited growth, and induced apoptosis in HT-29 and HCT-116 cells in a time- and dose-dependent manner [21].

One study found that the treatment of HT-29 cells with exogenous C16:0-Cer promoted the induction of cell death [22], which suggests that an increase in endogenous C16:0-Cer production could also lead to the same effects in RC, and a decrease in C16:0-Cer levels may result in the inhibition of apoptosis. At the same time, the authors compared the apoptotic response of HT-29 and HCT-116 cells and showed no effect of C16:0-Cer supplementation on the induction of cell death in the latter case, suggesting that this effect may be specific only to certain types of cells [22].

Further research is needed in this area. It should be emphasized that the interpretation of the obtained results is very difficult due to the high differentiation of tumor cells and tissues, additionally bearing in mind that both benign and malignant tissues can be present within colorectal tumors [23]. In addition, the metabolic pathways in cancer cells are very heterogeneous; as in colorectal cancer cells, some single enzymes involved in sphingolipid synthesis may have opposite effects on cell death in certain situations; some enzymes may be involved in two different pathways of ceramide production; and in addition, ceramides produced through two different synthetic pathways, may have different effects on cell survival [24].

The S1P/Cer ratio is an indicator of proliferative potential [25,26].

For each Cer, we calculated the S1P/Cer ratio and compared its value (colorectal cancer vs. healthy colorectal tissue), noting a significant increase in S1P/Cer in both cancer locations, as compared to healthy tissue. Differences were most visible for Cer with an 18-carbon chain length. The suppressive effect of high S1P/Cer on apoptotic signaling was first observed in HL-60 and U937 leukemia cell lines [27]. Regarding a particular Cer, pancreatic adenocarcinoma subclones which displayed increased proliferation were characterized by high S1P/C16:0-Cer ratios compared to an origin control cell line [28]; consistently with the S1P/Cer rheostat hypothesis, the inhibition of S1P synthesis reduced S1P/C16:0-Cer, inhibited proliferation, and increased apoptosis. Similar mechanisms regarding the increased viability of the cells were identified in an IMA-1 clone of the K562 leukemia cell line, where high S1P/C18:0-Cer was the main driving factor of resistance towards imatinib-induced cell death [29]. In the current study, we noted a profound increase in the S1P/Cer ratio for ceramides with an 18-carbon chain length, but not for palmitoyl-ceramide. Regarding colorectal cancer cells, resistance to cetuximab-induced cell death in various CRC cell lines was determined by the activity of sphingosine kinase 1 (SPHK1) and the S1P/Cer ratio [30].

In light of the above data, our results seem to prove that healthy rectal and colonic tissues have the same sphingolipid proliferative potential as determined by the S1P/Cer. Then, under the influence of unspecified carcinogenic stimuli, changes occur in the synthesis (or degradation) of specific Cer, leading to the current imbalance. In the large intestine (both rectum and colon), there is an increase in Sph levels in both locations. In addition, there are other changes in the content of sphingolipids, but their type and severity depend on the anatomical location. The S1P/Cer ratio was higher in cancer than in normal tissue for six Cer, although in smaller groups (RC and CC separately), statistically significant differences were observed for the four Cer in RC (S1P/C18:1-Cer, S1P/C20:0-Cer, S1P/C22:0-Cer, S1P/C24:1-Cer) and for three Cer in CC (S1P/C18:0-Cer, S1P/C18:1-Cer, S1P/C20:0-Cer).

It is generally accepted that most colorectal cancers develop from adenomas and are associated with acquired molecular events. One of the earlier studies confirmed the diverse trends of changes in the metabolism of S1P and Cer in the bowel, although it did not concern different anatomical locations, but various types of histological architecture and sundry grades of nuclear dysplasia. In tubular adenomas (with lower malignant potential), the amount of Cer increases compared with normal colorectal mucosa, whereas the content of S1P decreases; thus, the S1P/Cer amount was reduced [31]. Other relationships have been demonstrated in tubulovillous adenomas with high-grade dysplasia (and with higher malignant potential), where the Cer level decreased, while the S1P concentration increased; thus, the S1P/Cer amount was elevated [31].

Two earlier studies showed the highest C16:0-Cer content in both colorectal cancer and healthy colorectal tissue, and documented a tendency of various ceramide analogues to accumulate in CRC [3,8] and also in breast cancer [7] or in squamous cell carcinomas of the head and neck [32]. However, data on ceramide metabolism in CRC are still ambiguous and their mechanism has not been confirmed without doubt. In CRC, one study showed a higher content (compared to healthy adjacent tissue) of both S1P and several ceramide species [2].

Endogenous ceramides can be a product of sphingolipid degradation or de novo synthesis by serine palmitoyltransferase (SPT), ceramide synthase (CerS), and dihydroceramide desaturase (DES). Five CerS are expressed in intestinal mucosal cells producing ceramides with different acyl chain lengths; four CerS (1, 2, 5, 6) are dysregulated in CRC [24]. Most of the literature data show an increased expression of the CerS gene and the elevated content of ceramides in CRC [24]. CerS participates in the de novo synthesis and salvage production of ceramides, and it seems that accumulation of ceramides in colon cancer cells during apoptosis is a cancer-specific phenomenon, and ceramides generated from the de novo synthesis pathway are pro-survival, while those from sphingomyelinase hydrolyzation are pro-apoptotic [24]. Once formed, ceramides can accumulate or transform into other sphingolipids.

It is extremely interesting whether our observations will help explain the different courses of cancer in the future, and whether they will result in better individualized CRC treatments depending on the location of the tumor. The interrelationships regarding the SL content in cancer tissue are very intricate and certainly not fully understood, which should stimulate researchers to conduct further studies.

## 4. Materials and Methods

### 4.1. Patients

This study enrolled 30 consecutive patients admitted for elective colorectal surgery. This was a retrospective analysis of prospectively collected data, conducted in a single clinic at a university hospital. Written informed consent for participation in the study was obtained from all patients. The study was approved by the Ethical Committee for Human Studies of the Medical University of Bialystok and conducted in line with the principles stated in the Declaration of Helsinki; ethics committee approval no R-I-002/228/2018.

A pre-operative diagnosis of CRC was made based on colonoscopy and CT scan and confirmed by pathological examination. Tumor and adjacent tissue samples were obtained intraoperatively and immediately placed into liquid nitrogen. After surgical resection, both the tumor and the adjacent tissue were collected for examination and subjected to a full histopathological assessment to exclude neoplastic infiltration and confirm the resection’s radicality. Pathologic analysis of the resected specimens was made according to the American Joint Committee on Cancer criteria, version 8 guidelines.

### 4.2. Sphingolipid Content in Tissues

Sphingolipids were analyzed by ultra-high-performance liquid chromatography coupled with tandem mass spectrometry (triple quadrupole) (UHPLC/MS/MS) according to Blachnio-Zabielska et al. [33]. Tissue samples (20 mg) were pulverized and then homogenized in buffer (0.25 M sucrose, 25 mM KCl, 50 mM Tris, 0.5 mM EDTA, pH 7.4). Then, to each sample was added 50 μL of the internal standard mix (ISTD) Sph-d7, SPA-d7, S1P-d7, C15:0-d7-Cer, C16:0-d7-Cer, C18:1-d7-Cer, C18:0-d7-Cer, 17C20:0-Cer, C24:1-d7-Cer, C24-d7-Cer (Avanti Polar Lipids, Alabaster, AL, USA) and 2 mL of an extraction solution (isopropanol:water:ethyl acetate, 30:10:60; v:v:v). The samples were then mixed, sonicated, and centrifuged at 4000 rpm, at 4 °C for 10 min. (Sorvall Legend RT). The supernatant was transferred to new vials, the pellet was re-extracted and the resulting supernatant was combined with the previous one and evaporated under nitrogen. The dried samples were suspended in LC Solvent B (2 mM Ammonium formate, 0.1% formic acid in methanol) for UHPLC/MS/MS analysis. Sphingolipids: Sph (sphingosine), S1P (sphingosine-1-phosphate), SPA (sphinganine), ceramide C14:0-Cer (ceramide containing myristic acid), C16:0-Cer (ceramide containing palmitic acid), C18:1-Cer (ceramide containing oleic acid), C18:0-Cer (ceramide containing stearic acid), C20:0-Cer (ceramide containing arachidic acid), C22:0-Cer (ceramide containing behenic acid), C24:1-Cer (ceramide containing nervonic acid), and C24:0-Cer (ceramide containing lignoceric acid) were quantified using a Sciex Qtrap 6500 + mass spectrometer (SCIEX, Framingham, MA, USA) in a positive electrospray ionization (ESI) mode (except S1P, which was analyzed in negative mode) with multiple reaction monitoring (MRM) against standard curves constructed for each analyzed compound. Analyzed sphingolipids were separated on a reverse-phase Zorbax SB-C8 column 2.1 × 150 mm, 1.8 μm in a binary gradient (1 mM ammonium formate, 0.1% formic acid in water as Solvent A, and 2 mM ammonium formate, 0.1% formic acid in methanol as Solvent B, at a flow rate of 0.4 mL/min). The content of sphingosine-1-phosphate (S1P), sphinganine (SPA), sphingosine (Sph), and ceramides with saturated and unsaturated N-acyl chains of various lengths was determined.

### 4.3. Statistical Analysis

Comprehensive data were processed using Statistica 13.3 (TIBCO Software Inc., Palo Alto, CA, USA) and shown as the mean (M) ± standard error (SE). The Shapiro–Wilk test was used to assess the normality of the data. The mean values were compared using a paired t-student test. Statistical significance was assumed if a *p*-value was less than 0.05.

## 5. Conclusions

It seems that the development and progression of colorectal cancer are accompanied by complex changes in the metabolism of sphingolipids, causing not only qualitative shifts in the ceramide pool of cancer tissue but also quantitative disturbances, depending on the location of the primary tumor. In the current study, we have shown for the first time that the sphingolipid profile is differentiated in rectal and colon cancer. The lower content of ceramides in rectal cancer may be responsible for its aggressive course and is an incentive to consider the use of exogenous ceramides, ceramide-generating agents, or modulators of ceramide metabolism in rectal cancer combination therapy as an individualized oncological treatment.

## Figures and Tables

**Table 1:** The content of sphingolipids in colorectal tumor tissue (also divided into rectal and colonic) and healthy, adjacent tissue. Statistical significance is marked with an asterisk. Values expressed as [pmol/mg] are presented as mean ± standard error of mean [M ± SE]; * *p* < 0.05. The direction of the arrow indicates a significant increase or decrease.

**1A. Sphingolipids** **in Colorectal** **Cancer (CRC).**	**Adjacent Tissue** **(A). n = 30.**	**Tumor Tissue** **(T). n = 30.**	**T vs. A**
	**M ± SE**	**M ± SE**	***p***
**Sph**	2.38 ± 0.22	↑ 6.09 ± 0.73	0.0001 *
**S1P**	0.02 ± 0.001	↑ 0.05 ± 0.01	0.0006 *
**SPA**	0.70 ± 0.1	↑ 1.37 ± 0.14	0.0003 *
**C14:0-Cer**	1.17 ± 0.1	↑ 1.66 ± 0.13	0.0031 *
**C16:0-Cer**	106.33 ± 8.09	126.75 ± 10.19	0.1220
**C18:1-Cer**	0.30 ± 0.02	0.31 ± 0.03	0.8100
**C18:0-Cer**	3.91 ± 0.26	3.21 ± 0.21 ↓	0.0366 *
**C20:0-Cer**	1.29 ± 0.09	0.92 ± 0.08 ↓	0.0020 *
**C22:0-Cer**	3.11 ± 0.16	3.12 ± 0.22	0.9764
**C24:1-Cer**	13.53 ± 0.85	14.00 ± 0.96	0.7150
**C24:0-Cer**	4.69 ± 0.27	↑ 6.41 ± 0.48	0.0027 *
**Total Cer**	134.34 ± 9.18	156.38 ± 11.23	0.1341
**1B. Sphingolipids in rectal** **cancer (RC).**	**Adjacent tissue** **(A). n = 16.**	**Tumor tissue** **(T). n = 16.**	**T vs. A**
	**M ± SE**	**M ± SE**	***p***
**Sph**	2.38 ± 0.34	↑ 5.16 ± 0.85	0.0050 *
**S1P**	0.03 ± 0.01	0.04 ± 0.01	0.1244
**SPA**	0.69 ± 0.18	1.05 ± 0.15	0.1343
**C14:0-Cer**	1.11 ± 0.14	1.43 ± 0.17	0.1656
**C16:0-Cer**	100.16 ± 11.42	107.73 ± 13.0	0.6646
**C18:1-Cer**	0.30 ± 0.04	0.29 ± 0.04	0.7525
**C18:0-Cer**	3.96 ± 0.39	2.92 ± 0.21 ↓	0.0270 *
**C20:0-Cer**	1.37 ± 0.12	0.91 ± 0.11 ↓	0.0091 *
**C22:0-Cer**	3.18 ± 0.22	2.56 ± 0.24	0.0712
**C24:1-Cer**	13.90 ± 1.26	14.08 ± 1.62	0.9306
**C24:0-Cer**	4.67 ± 0.38	5.17 ± 0.6	0.4839
**Total Cer**	128.66 ± 13.09	135.10 ± 14.78	0.7463
**1C. Sphingolipids in colon** **cancer (CC).**	**Adjacent tissue** **(A). n = 14.**	**Tumor tissue** **(T). n = 14.**	**T vs. A**
	**M ± SE**	**M ± SE**	***p***
**Sph**	2.39 ± 0.26	↑ 7.17 ± 1.19	0.0006 *
**S1P**	0.02 ± 0.001	↑ 0.06 ± 0.01	0.0011 *
**SPA**	0.72 ± 0.07	↑ 1.73 ± 0.21	0.0001 *
**C14:0-Cer**	1.23 ± 0.13	↑ 1.92 ± 0.16	0.0030 *
**C16:0-Cer**	113.38 ± 11.58	148.48 ± 14.32	0.0678
**C18:1-Cer**	0.30 ± 0.03	0.34 ± 0.04	0.3921
**C18:0-Cer**	3.86 ± 0.33	3.53 ± 0.36	0.5112
**C20:0-Cer**	1.20 ± 0.13	0.93 ± 0.10	0.1066
**C22:0-Cer**	3.03 ± 0.25	3.76 ± 0.31	0.0783
**C24:1-Cer**	13.10 ± 1.15	13.91 ± 0.96	0.5980
**C24:0-Cer**	4.71 ± 0.39	↑ 7.82 ± 0.57	0.0001 *
**Total Cer**	140.83 ± 13.07	180.69 ± 15.15	0.0570

**Table 2:** The content of sphingolipids in colorectal tumor tissue (also divided into rectal and colonic). Statistical significance is marked with an asterisk. Values expressed as [pmol/mg] are presented as mean ± standard error of mean [M ± SE]; * *p* < 0.05. The direction of the arrow indicates a significant increase or decrease.

**2A. Sphingolipids in Tumor** **Tissue (T)**	**Rectal Cancer** **(RC). n = 16.**	**Colon Cancer** **(CC). n = 14.**	**RC vs. CC**
	**M ± SE**	**M ± SE**	***p***
**Sph**	5.16 ± 0.85	7.17 ± 1.19	0.172
**S1P**	0.04 ± 0.01	0.06 ± 0.01	0.162
**SPA**	1.05 ± 0.15 ↓	1.73 ± 0.21	0.013 *
**C14:0-Cer**	1.43 ± 0.17	1.92 ± 0.16	0.053
**C16:0-Cer**	107.73 ± 13.00 ↓	148.48 ± 14.32	0.044 *
**C18:1-Cer**	0.29 ± 0.04	0.34 ± 0.04	0.312
**C18:0-Cer**	2.92 ± 0.21	3.53 ± 0.36	0.151
**C20:0-Cer**	0.91 ± 0.11	0.93 ± 0.10	0.879
**C22:0-Cer**	2.56 ± 0.24 ↓	3.76 ± 0.31	0.005 *
**C24:1-Cer**	14.08 ± 1.62	13.91 ± 0.96	0.928
**C24:0-Cer**	5.17 ± 0.60 ↓	7.82 ± 0.57	0.004 *
**Total Cer**	135.10 ± 14.78 ↓	180.69 ± 15.15	0.041 *
**2B. Sphingolipids in adjacent** **tissue (A)**	**Rectal cancer** **(RC). n = 16.**	**Colon cancer** **(CC). n = 14.**	**RC vs. CC**
	**M ± SE**	**M ± SE**	***p***
**Sph**	2.38 ± 0.34	2.39 ± 0.26	0.971
**S1P**	0.03 ± 0.01	0.02 ± 0.00	0.400
**SPA**	0.69 ± 0.18	0.72 ± 0.07	0.861
**C14:0-Cer**	1.11 ± 0.14	1.23 ± 0.13	0.547
**C16:0-Cer**	100.16 ± 11.42	113.38 ± 11.58	0.425
**C18:1-Cer**	0.30 ± 0.04	0.30 ± 0.03	1.000
**C18:0-Cer**	3.96 ± 0.39	3.86 ± 0.33	0.847
**C20:0-Cer**	1.37 ± 0.12	1.20 ± 0.13	0.344
**C22:0-Cer**	3.18 ± 0.22	3.03 ± 0.25	0.661
**C24:1-Cer**	13.90 ± 1.26	13.10 ± 1.15	0.647
**C24:0-Cer**	4.67 ± 0.38	4.71 ± 0.39	0.939
**Total Cer**	128.66 ± 13.09	140.83 ± 13.07	0.518

**Table 3:** The content of S1P/Cer in colorectal tumor tissue (also divided into rectal and colonic) and healthy, adjacent tissue. Statistical significance is marked with an asterisk. Values expressed as [pmol/mg] are presented as mean ± standard error of mean [M ± SE]; * *p* < 0.05. The direction of the arrow indicates a significant increase or decrease.

**3A. S1P/Cer** **in Colorectal** **Cancer (CRC).**	**Adjacent Tissue** **(A). n = 30.**	**Tumor Tissue** **(T). n = 30.**	**T vs. A**
	**M ± SE**	**M ± SE**	***p***
S1P/C14:0-Cer	0.0275 ± 0.007	0.0378 ± 0.007	0.2899
S1P/C16:0-Cer	0.0004 ± 0.001	0.0005 ± 0.001	0.3157
S1P/C18:1-Cer	0.0070 ± 0.001	↑ 0.2202 ± 0.048	0.0000 *
S1P/C18:0-Cer	0.0081 ± 0.002	↑ 0.0200 ± 0.005	0.0241 *
S1P/C20:0-Cer	0.0219 ± 0.004	↑ 0.0715 ± 0.015	0.0018 *
S1P/C22:0-Cer	0.0085 ± 0.002	↑ 0.0184 ± 0.003	0.0081 *
S1P/C24:1-Cer	0.0020 ± 0.001	↑ 0.0044 ± 0.001	0.0081 *
S1P/C24:0-Cer	0.0055 ± 0.001	↑ 0.0089 ± 0.001	0.0499 *
S1P/TCer	0.0002 ± 0.001	0.0004 ± 0.001	0.0769
**3B. S1P/Cer** **in rectal** **cancer (RC).**	**Adjacent tissue** **(A). n = 16.**	**Tumor tissue** **(T). n = 16.**	**T vs. A**
	**M ± SE**	**M ± SE**	***p***
S1P/C14:0-Cer	0.0338 ± 0.011	0.0450 ± 0.008	0.5075
S1P/C16:0-Cer	0.0005 ± 0.001	0.0007 ± 0.001	0.3813
S1P/C18:1-Cer	0.0077 ± 0.002	↑ 0.2885 ± 0.066	0.0023 *
S1P/C18:0-Cer	0.0105 ± 0.004	0.0251 ± 0.007	0.1199
S1P/C20:0-Cer	0.0233 ± 0.006	↑ 0.0920 ± 0.024	0.0122 *
S1P/C22:0-Cer	0.0093 ± 0.002	↑ 0.0224 ± 0.006	0.0337 *
S1P/C24:1-Cer	0.0022 ± 0.001	↑ 0.0054 ± 0.001	0.0340 *
S1P/C24:0-Cer	0.0061 ± 0.001	0.0107 ± 0.002	0.0967
S1P/TCer	0.0003 ± 0.001	0.0005 ± 0.001	0.1545
**3C. S1P/Cer** **in colon** **cancer (CC).**	**Adjacent tissue** **(A). n = 14.**	**Tumor tissue** **(T). n = 14.**	**T vs. A**
	**M ± SE**	**M ± SE**	***p***
S1P/C14:0-Cer	0.0204 ± 0.006	0.0296 ± 0.012	0.2339
S1P/C16:0-Cer	0.0003 ± 0.001	0.0003 ± 0.001	0.5820
S1P/C18:1-Cer	0.0062 ± 0.001	↑ 0.1421 ± 0.072	0.0000 *
S1P/C18:0-Cer	0.0054 ± 0.001	↑ 0.0142 ± 0.006	0.0089 *
S1P/C20:0-Cer	0.0204 ± 0.005	↑ 0.0482 ± 0.016	0.0299 *
S1P/C22:0-Cer	0.0075 ± 0.002	0.0138 ± 0.003	0.1028
S1P/C24:1-Cer	0.0018 ± 0.001	0.0033 ± 0.001	0.0984
S1P/C24:0-Cer	0.0048 ± 0.001	0.0069 ± 0.002	0.2983
S1P/TCer	0.0002 ± 0.001	0.0003 ± 0.001	0.1485
**3D. S1P/Cer** **in tumor** **tissue (T)**	**Rectal cancer** **(RC). n = 16.**	**Colon cancer** **(CC). n = 14.**	**RC vs. CC**
	**M ± SE**	**M ± SE**	***p***
S1P/C14:0-Cer	0.0450 ± 0.008	0.0296 ± 0.012	0.2798
S1P/C16:0-Cer	0.0007 ± 0.001	0.0003 ± 0.001	0.1122
S1P/C18:1-Cer	0.2885 ± 0.066	0.1421 ± 0.072	0.1300
S1P/C18:0-Cer	0.0251 ± 0.007	0.0142 ± 0.006	0.2340
S1P/C20:0-Cer	0.0920 ± 0.024	0.0482 ± 0.016	0.1383
S1P/C22:0-Cer	0.0224 ± 0.006	0.0138 ± 0.003	0.1989
S1P/C24:1-Cer	0.0054 ± 0.001	0.0033 ± 0.001	0.1989
S1P/C24:0-Cer	0.0107 ± 0.002	0.0069 ± 0.002	0.2057
S1P/TCer	0.0005 ± 0.001	0.0003 ± 0.001	0.1211
**3E. S1P/Cer** **in adjacent** **tissue (A)**	**Rectal cancer** **(RC). n = 16.**	**Colon cancer** **(CC). n = 14.**	**RC vs. CC**
	**M ± SE**	**M ± SE**	***p***
S1P/C14:0-Cer	0.0338 ± 0.011	0.0204 ± 0.006	0.3131
S1P/C16:0-Cer	0.0005 ± 0.001	0.0003 ± 0.001	0.4654
S1P/C18:1-Cer	0.0077 ± 0.002	0.0062 ± 0.001	0.4908
S1P/C18:0-Cer	0.0105 ± 0.004	0.0054 ± 0.001	0.3067
S1P/C20:0-Cer	0.0233 ± 0.006	0.0204 ± 0.005	0.7263
S1P/C22:0-Cer	0.0093 ± 0.002	0.0075 ± 0.002	0.5505
S1P/C24:1-Cer	0.0022 ± 0.001	0.0018 ± 0.001	0.5464
S1P/C24:0-Cer	0.0061 ± 0.001	0.0048 ± 0.001	0.4952
S1P/TCer	0.0003 ± 0.001	0.0002 ± 0.001	0.3662

## Data Availability

The data presented in this study are available on request from the corresponding author.
